# A novel anionic-phosphate-platinum complex effectively targets an undifferentiated pleomorphic sarcoma better than cisplatinum and doxorubicin in a patient-derived orthotopic xenograft (PDOX)

**DOI:** 10.18632/oncotarget.18806

**Published:** 2017-06-28

**Authors:** Kentaro Igarashi, Kei Kawaguchi, Takashi Murakami, Tasuku Kiyuna, Kentaro Miyake, Norio Yamamoto, Katsuhiro Hayashi, Hiroaki Kimura, Scott D. Nelson, Sarah M. Dry, Yunfeng Li, Arun S. Singh, Shinji Miwa, Akira Odani, Fritz C. Eilber, Hiroyuki Tsuchiya, Robert M. Hoffman

**Affiliations:** ^1^ AntiCancer, Inc., San Diego, California, USA; ^2^ Department of Surgery, University of California, San Diego, California, USA; ^3^ Department of Orthopaedic Surgery, Kanazawa University, Kanazawa, Japan; ^4^ Department of Pathology, University of California, Los Angeles, California, USA; ^5^ Division of Hematology-Oncology, University of California, Los Angeles, California, USA; ^6^ Division of Pharmaceutical Sciences, Kanazawa University, Kanazawa, Japan; ^7^ Division of Surgical Oncology, University of California, Los Angeles, California, USA

**Keywords:** platinum complex, 3Pt, undifferentiated pleomorphic sarcoma, PDOX, efficacy

## Abstract

A patient high-grade undifferentiated pleomorphic soft-tissue sarcoma (UPS) from a striated muscle was previously orthotopically implanted in the right biceps femoris muscle of nude mice to establish a patient-derived orthotopic xenograft (PDOX) nude-mouse model. In the present study, two weeks after orthotopic transplantation of the UPS, mice were treated intraperitoneally with cisplatinum (CDDP), doxorubicin (DOX) or a novel anionic-phosphate-platinum compound 3Pt. Treatments were repeated weekly for a total of 3 times. Six weeks after transplantation, all mice were sacrificed and evaluated. After two weeks treatment, tumor sizes were as follows: control (G1): 2208.3 mm^3^; CDDP (G2): 841.8±3 mm^3^, p=0.0001; DOX (G3): 693.1±3 mm^3^, p=6.56E-7; 3Pt (G4): 333.7±1 mm^3^, p=4.8E-8. 3Pt showed significantly more efficacy compared to other therapy drugs tested: CDDP (p=0.0002), DOX (p=0.001). There were no animal deaths in any of the four groups. The present results suggest 3Pt is a promising new candidate for UPS since it was demonstrated to be effective in a PDOX model.

## INTRODUCTION

Our laboratory pioneered patient-derived orthotopic xenograft (PDOX) mouse models of cancer using surgical orthotopic implantation (SOI). PDOX models are patient-like, in contrast to the ectopic subcutaneous-transplant cancer models [1]. In a previous study, a patient with high-grade undifferentiated pleomorphic soft-tissue sarcoma (UPS) from a striated muscle was transplanted orthotopically in the right biceps femoris muscle of mice to establish a UPS PDOX model. Histological analysis indicated tumor eradication only when mice were treated with tumor-targeting *S. typhimurium* A1-R followed by doxorubicin (DOX) [2].

We previously developed a novel platinum complex, 3Pt. 3Pt comprises anionic-phosphate moieties. The cytotoxic potency of 3Pt was greater than that of cisplatinum (CDDP) in all osteosarcoma cell lines tested. 3Pt was not cross resistant in CDDP-resistant cells [3].

In the present study, we determined the efficacy of 3Pt against the UPS PDOX model for comparison to DOX and CDDP.

## RESULTS AND DISCUSSION

### Drug efficacy

**Figure 1:**
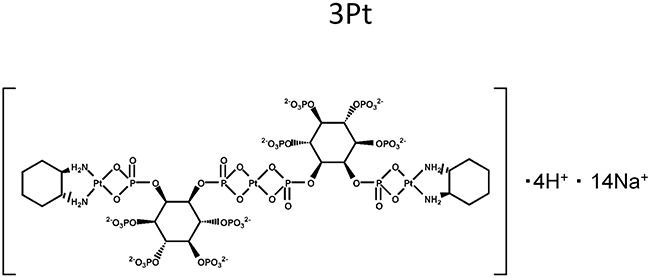
Chemical structure of the novel platinum compound 3Pt

**Figure 2:**
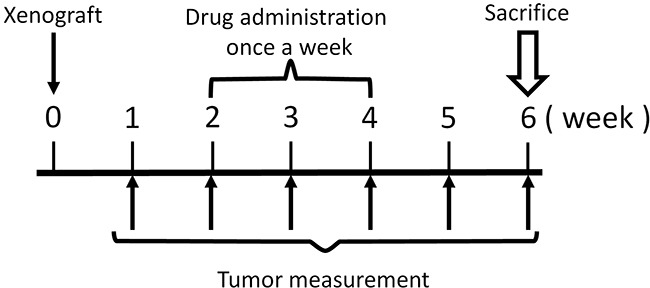
Treatment schema

**Figure 3:**
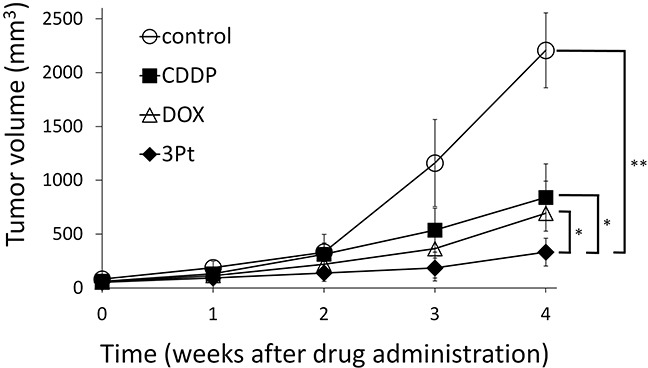
Efficacy of cisplatin (CDDP), doxorubicin (DOX), and 3Pt on the UPS PDOX nude-mouse models DOX (3 mg/kg, i.p., qw×3); CDDP (6 mg/kg, i.p., qw×3); 3Pt (41.1 mg/kg, i.p., qw×3). Tumor volume was measured at the indicated time points after the onset of treatment. n=8 mice/group; * p < 0.005, ** p<0.0001.

**Figure 4:**
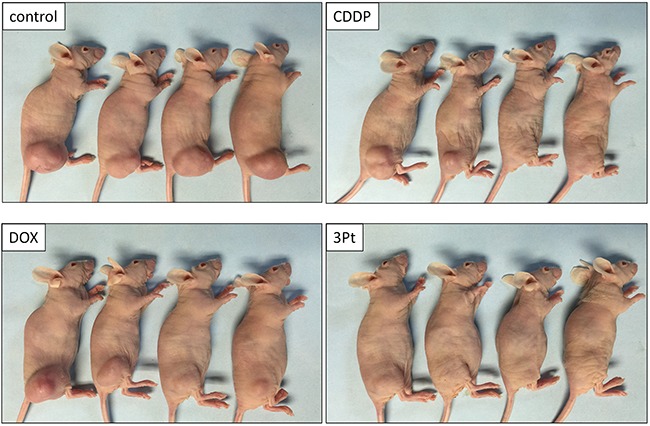
Efficacy of CDDP, DOX and 3Pt on the UPS PDOX Photographs of representative PDOX nude-mouse models in each treatment group at the end of the treatment period.

**Figure 5:**
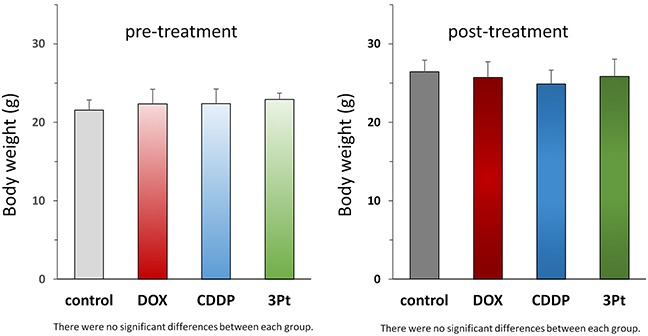
Body weight of treated and untreated mice Bar graphs show body weight in each group at pre-treatment and 4 weeks after drug administration.

The novel anionic-phosphate-platinum complex 3Pt (Figure 1) was evaluated in the schema shown in (Figure 2). Two weeks after orthotopic implantation, UPS tumors grew to 5 mm in diameter in PDOX nude-mouse models. Thirty-two PDOX mice were randomized into 4 groups: untreated controls; DOX; CDDP; and 3Pt. Treatments were repeated once a week for 3 weeks. Six weeks after transplantation, all mice were sacrificed. At 6 weeks, treatment of the UPS PDOX with 3Pt resulted in the largest decrease in tumor volume relative to untreated controls compared to DOX or CDDP: Control (G1): 2208.3 mm^3^; CDDP (G2): 841.8 mm^3^, p=0.0001; DOX (G3): 693.1 mm^3^, p = 6.56E-7; 3Pt (G4): 333.7 mm^3^, p = 4.8E-8. 3Pt showed significantly more efficacy compared to CDDP (p = 0.0002) or DOX (p = 0.001) (Figures 3, 4). There were no animal deaths in any of the four groups. Body weights of the treated mice were not significantly different from untreated controls in any group (Figure 5).

### Histology of original tumor and implanted tumors

**Figure 6:**
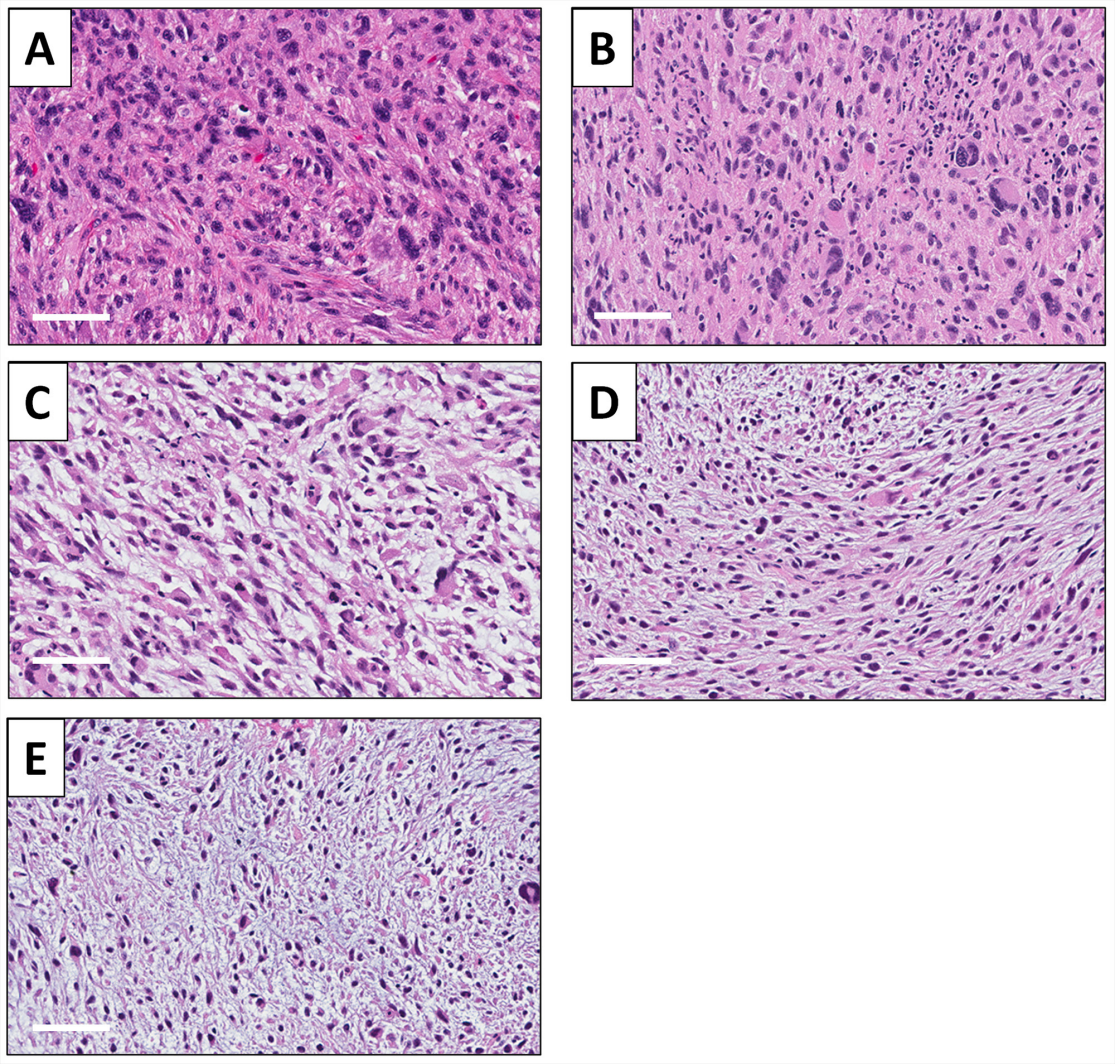
Tumor histology **(A)** Hematoxylin and eosin (H&E)-stained sections of the original patient tumor. **(B)** Untreated UPS PDOX tumor. **(C)** UPS PDOX tumor treated with CDDP. **(D)** UPS PDOX tumor treated with DOX. **(E)** UPS PDOX tumor treated with 3Pt. Scale bars: 80 μm.

A high-power photomicrograph of the original patient tumor hematoxylin and eosin (H&E)-stained section showed solid sheets of cancer cells by various shapes, including spindle-shaped, round-shaped with hyperchromatic, enlarged nuclei as well as bizarre multinucleate giant cells. Numerous mitotic figures, including atypical forms are present (Figure 6A). The high-power view of the H&E section of the untreated PDOX tumor had similar features including spindle-shaped cells and round-shaped cells. Numerous mitotic figures, including atypical forms are also present (Figure 6B). PDOX tumors treated with CDDP were comprised of various shaped viable cells without apparent necrosis (Figure 6C). PDOX tumors treated with DOX had viable cells with little necrosis (Figure 6D). The 3Pt-treated PDOX tumor showed apparent necrosis and fibrosis (Figure 6E).

## MATERIALS AND METHODS

### Animal care

Athymic nu/nu nude mice (AntiCancer Inc., San Diego, CA), 4–6 weeks old, were used in this study. Animals were housed in a barrier facility on a high efficiency particulate arrestance (HEPA) - filtered rack under standard conditions of 12-hour light/dark cycles. The animals were fed an autoclaved laboratory rodent diet. All animal studies were conducted with an AntiCancer Institutional Animal Care and Use Committee (IACUC)-protocol specifically approved for this study and in accordance with the principals and procedures outlined in the National Institutes of Health Guide for the Care and Use of Animals under Assurance Number A3873-1. In order to minimize any suffering of the animals the use of anesthesia and analgesics were used for all surgical experiments. Animals were anesthetized by subcutaneous injection of a 0.02 ml solution of 20 mg/kg ketamine, 15.2 mg/kg xylazine, and 0.48 mg/kg acepromazine maleate. The response of animals during surgery was monitored to ensure adequate depth of anesthesia. The animals were observed on a daily basis and humanely sacrificed by CO_2_ inhalation when they met the following humane endpoint criteria: severe tumor burden (more than 20 mm in diameter), prostration, significant body weight loss, difficulty breathing, rotational motion and body temperature drop.

### Patient-derived tumor

A 64-year-old male diagnosed with high-grade undifferentiated pleomorphic sarcoma (UPS) on his right high thigh previously underwent surgical resection at the Department of Surgery, University of California, Los Angeles (UCLA), under written informed consent and IRB approval (IRB #10-001857). The patient did not receive any chemotherapy or radiotherapy prior to surgery. A part of the resected specimen, designated as PDOX1, was sent to our laboratory right after surgery and implanted in nude mice [2, 4].

### Drugs

The novel platinum drug, 3Pt (Figure 1) was synthesized as previously described [3]. CDDP and DOX were purchased from Teva Parenteral Medicine, Inc. (Irvine, CA).

### Surgical orthotopic implantation (SOI) for establishment of the PDOX model

After subcutaneous growth of the patient tumor in nude mice, it was harvested and divided into small fragments (3-4 mm) for orthotopic transplantation in nude mice. After the nude mice were anesthetized, a 5 mm skin incision was made on the right high thigh. The biceps femoris was then split to make space for a tumor fragment. A single tumor fragment was implanted orthotopically into the space to establish a PDOX model. The wound was closed with 6-0 nylon suture (Ethilon, Ethicon, Inc., NJ, USA).

### Treatment study design

Two weeks after orthotopic implantation, tumors reached 5 mm in diameter. Thirty-two UPS PDOX mice were randomized into 4 groups (Figure 2): G1: control without treatment (n=8); G2: cisplatin (CDDP) (6 mg/kg), intraperitoneal (i.p.) injection, weekly for 3 weeks) (n=8); G3: Doxorubicin (DOX) (3 mg/kg, i.p. injection, weekly for 3 weeks) (n=8); G4: 3Pt (41.1mg/kg), i.p. injection, weekly for 3 weeks) (n=8). Tumor length, width, and mouse body weight were measured once a week. Tumor volume was calculated with the following formula: Tumor volume (mm^3^) = length (mm) × width (mm) × width (mm) × 1/2. Data are presented as mean ± SD. All treated mice were sacrificed after 6 weeks and tumors were resected for histological analysis (please see below).

### Histology

Fresh tumor samples were fixed in 10% formalin and embedded in paraffin before sectioning and staining. Tissue sections (3 μm) were deparaffinized in xylene and rehydrated in an ethanol series. H&E staining was performed according to a standard protocol. Histological examination was performed with a BHS system microscope. Images were acquired with INFINITY ANALYZE software (Lumenera Corporation, Ottawa, Canada).

### Statistical analysis

Data are presented as means±standard deviation and were compared between groups using the unpaired Student's *t-*test. A *p<*0.05 value was considered statistically significant.

## CONCLUSION

In the present study, we compared a novel anionic-phosphate-platinum compound; 3Pt, with CDDP and DOX against an UPS PDOX nude mouse model. 3Pt showed significantly more efficacy compared to CDDP (p=0.0002) or DOX (p=0.001) which are first-line therapy for this disease. 3Pt is a promising candidate for UPS since it was effective in a PDOX model. Future studies will combine 3Pt with other drugs on UPS and other sarcoma PDOX models in order to identify regimens which eradicate tumors and are therefore of high potential in the clinic.

Toward the goal of personalized, precision oncology, our laboratory pioneered the patient-derived orthotopic xenograft (PDOX) nude mouse model with the technique of surgical orthotopic implantation (SOI), including pancreatic [5–8], breast [9], ovarian [10], lung [11], cervical [12], colon [13–15], stomach [16], sarcoma [2, 17–20] and melanoma [21–24]. The present manuscript demonstrates the precision with which the UPS PDOX model distinguishes responses to CDDP, DOX, and 3Pt.

Previously-developed concepts and strategies of highly-selective tumor targeting can take advantage of molecular targeting of tumors, including tissue-selective therapy which focuses on unique differences between normal and tumor tissues [25–30].
